# Enhancement of Disease Resistance in Pengze Crucian Carp (*Carassius auratus var. Pengze*) by Carvacrol Through Modulation of Intestinal Microbiota and Serum Metabolism

**DOI:** 10.3390/metabo16030151

**Published:** 2026-02-25

**Authors:** Yuzhu Wang, Xiaoze Guo, Jingjing Lu, Lingya Li, Yanqiang Tang, Haihong Xiao, Siming Li, Wenshu Liu

**Affiliations:** 1 Institute of Animal Husbandry and Veterinary, Jiangxi Academy of Agricultural Sciences, Nanchang 330200, China; wangyuzhu@jxaas.cn (Y.W.); guoxz@jxaas.cn (X.G.); lujingjing@jxaas.cn (J.L.); lilingya@jxaas.cn (L.L.); tangyqq@jxaas.cn (Y.T.); xiaohh@jxaas.cn (H.X.); lisiming@jxaas.cn (S.L.); 2 Jiangxi Province Key Laboratory of Animal Green and Healthy Breeding, Nanchang 330200, China

**Keywords:** carvacrol, Pengze crucian carp, microbiome, metabolome, disease resistance

## Abstract

**Highlights:**

**What are the main findings?**

- Microencapsulated carvacrol at 600 mg/kg reduces the abundance of intestinal pathogenic bacteria and increases the levels of beneficial bacteria such as *Cetobacterium* in Pengze crucian carp.
- Carvacrol modulates metabolites in serum lipid metabolism, autophagy and other related pathways.

**What are the implications of the main findings?**

- This study provides a research reference for the effects of carvacrol on intestinal microbiota and serum metabolism in fish.
- It offers experimental evidence for the application of plant-derived immunostimulants as antibiotic alternatives in the green aquaculture of freshwater fish.

**Abstract:**

**Objectives:** This study aimed to investigate the regulatory effects of dietary carvacrol on intestinal micro biota composition, serum metabolic profiles, and their association with increased resistance to *Aeromonas hydrophila* in Pengze crucian carp. **Methods:** Juvenile fish (5.63 ± 0.35 g) were randomly allocated into two experimental groups: a control group (CK) fed a basal diet and a treatment group (CA) supplemented with 600 mg/kg microencapsulated carvacrol. Following an 8-week feeding trial, nine specimens per group were sampled for venous blood and intestinal tract collection. Remaining individuals were subjected to a 12-h *A. hydrophila* challenge prior to identical sample collection. **Results:** Key findings revealed that carvacrol supplementation induced significant microbial modulations, notably reducing Firmicutes abundance while enhancing *Cetobacterium* populations by 33.25% compared to controls. Post-challenge analysis demonstrated marked declines in intestinal microbial diversity indices (Observed ASV, Chao1, ACE, and PD whole tree) in the CK group, whereas the CA group maintained stable microbial diversity. Pathogenic genera including *Aeromonas*, *Shewanella*, and *Vibrio* showed significant proliferation in challenged controls, contrasting with maintained microbial homeostasis in carvacrol-fed specimens. Serum metabolomic profiling identified the most significantly altered metabolic pathways associated with carvacrol administration: glycerophospholipid metabolism, linoleic acid metabolism, arachidonic acid metabolism, α-linolenic acid metabolism, GPI-anchor biosynthesis, and autophagy-animal pathways. **Conclusions:** Our results demonstrate that dietary carvacrol may reinforce intestinal microbial barrier function by optimizing beneficial microbial composition and reducing the proportion of pathogens, and modulate immune-related metabolic pathways critical for host defense, which might be involved in enhanced disease resistance.

## 1. Introduction

Antibiotics have been widely employed as feed additives for decades, given their ability to enhance feed conversion efficiency and modify the immune status of animals by regulating gastrointestinal diseases and intestinal microbiota [1]. However, the widespread use of antibiotics has elicited significant concerns within the scientific community [2]. The incomplete absorption of veterinary antibiotics, which results in their excretion into aquatic and soil environments [3], fuels the emergence and proliferation of antibiotic-resistant bacteria (ARB) [4], making ARB a pervasive global health challenge. In recent years, many nations and organizations have moved to ban the use of antibiotics in livestock farming [5,6,7]. However, this reduction has had its drawbacks: reduced animal productivity and higher mortality rates [8]. Therefore, maintaining animal growth performance without resorting to antibiotic additives has emerged as a critical area of research in the livestock industry, and the search for an effective “immunostimulant” alternative with superior antibacterial properties seems to offer a promising solution to this dilemma [9,10].

The utilization of “immunostimulants” has been established as an effective approach to enhance the immune status of animal organisms and prevent the outbreak of diseases. Most of them are considered to have low toxicity, and are also capable of bolstering immune functions [11], which has made them one of the most vibrant research areas in feed hygiene, health maintenance, and feed additive development. Carvacrol, chemically known as 5-isopropyl-2-methylphenol, is the primary active constituent in oregano oil. Widely regarded as an excellent “immunostimulant”, carvacrol has been reported in numerous studies to confer various benefits to fish when administered either as a standalone compound or in blends, including antibacterial effects [12], growth promotion [13], and enhancement of immune functions [14,15].

Pengze crucian carp (*Carassius auratus var. Pengze*), belonging to the *Cyprinidae* family and the *Carassius* genus, is a crucian carp variety selectively bred by the Jiangxi Provincial Fisheries Science Research Institute and the Jiujiang Fisheries Science Research Institute in the 1980s. Renowned for its succulent flesh, nutritional value, and production capabilities, Pengze crucian carp stands as a premium species for large-scale artificial aquaculture in China. Nevertheless, the prevalent practice of high-density intensive farming in the country exposes Pengze crucian carp to a significant potential risk of disease outbreaks.

Numerous studies have indicated that carvacrol exerts significant regulatory effects on fish intestinal health [16], metabolic substances [17], and immune responses [18]. Furthermore, our preliminary research demonstrated that supplementing feed with 600 mg/kg of carvacrol can improve intestinal morphology and alleviate intestinal inflammation in Pengze crucian carp [19]. Therefore, building upon our previous research, leveraging microbiome and metabolome approaches, we planned and conducted subsequent experiments to investigate the dynamic changes in intestinal microbiota and the metabolic pathways in response to carvacrol exposure in Pengze crucian carp, and our goal is to provide a reference for the development and application of carvacrol as an antibiotic alternative in aquatic animals.

## 2. Materials and Methods

### 2.1. Experimental Diets

The formula and components of the experimental feed are shown in Table 1. According to our previous experimental method and results [19], all feed ingredients were crushed into powder that could pass through a 200-mesh sieve, and then 0 or 600 mg/kg of microencapsulated carvacrol (10%) was added to the feed and thoroughly blended: the Control Check (CK) group received no addition, whereas the Carvacrol Addition (CA) group was supplemented with 600 mg/kg of microencapsulated carvacrol. The feed blend was then processed into pellets using a manual feed pellet machine, followed by air-drying at ambient temperatures ranging from 24 to 31 °C. Subsequently, the large pellets were crushed into small particles, and appropriately sized particles were collected through sieving (40-mesh). Finally, the prepared experimental feed was stored in a −20 °C refrigerator for future use.

### 2.2. Fish and Feeding Management

The juvenile Pengze crucian carp used in the present study were obtained from a breeding base located in Jiujiang City, Jiangxi Province. Before the formal feeding experiment, the fish were acclimated for two weeks in the aquaculture laboratory of the Jiangxi Academy of Agricultural Sciences. Both the acclimation and feeding phases were performed in aquariums (60.0 cm × 60.0 cm × 80.0 cm) with a recirculating filtration system. A total of six aquariums were used in this experiment, with three aquariums assigned to each experimental group. Ten fish of uniform size (5.63 ± 0.35 g) were randomly distributed into each aquarium. The feeding trial lasted for 8 weeks, during which the fish were manually fed to apparent satiation twice daily (at 09:00 and 15:00). The initial feeding rate was set at 3% of the fish’s body weight and adjusted accordingly based on their feeding behavior. Throughout the experiment, one-third of the water in each aquarium was replaced daily with isothermal aerated water, and continuous aeration was provided using an air pump. Daily water quality monitoring was performed to ensure that the following conditions were maintained: pH (6.5–7.6), dissolved oxygen (>6.80 mg/L), temperature (27.8 ± 0.9 °C), and nitrate (<0.08 mg/L). After the feeding trial, 10 fish from each group were randomly selected to undergo a 12-h immersion challenge test with *A. hydrophila*. The *A. hydrophila* strain used was NJ-1, generously provided by Dr. Zhou from the Institute of Feed Research of Chinese Academy of Agricultural Sciences. Based on previous research findings [20], the concentration of *A. hydrophila* utilized was 1 × 10^8^ cells/mL.

### 2.3. Sample Collection and Preparation

Upon completion of the 8-week feeding trial, all fish groups in the experimental sets underwent a 24-h fasting period. Following this, 10 fish were randomly selected from each group for blood sampling via the caudal vein. The blood samples were left to stand overnight at 4 °C, then centrifuged at 3000 rpm for 10 min to separate the serum. The resulting supernatant was transferred into sterile polyethylene (PE) tubes and stored in a −80 °C freezer for subsequent testing. After blood collection, the fish were humanely euthanized by quickly disrupting their brain tissue using sharp-tipped forceps. Under strict aseptic conditions, the fish were dissected to extract the entire intestinal tissue, which was placed in sterile, enzyme-free PE tubes and stored in a −80 °C freezer for further analysis. The procedures for collecting intestinal tissue and serum samples after the 12-h immersion challenge test with Pengze crucian carp were identical to the above-mentioned process.

### 2.4. 16S rRNA Sequence Determination of Intestinal Microorganisms

16S rDNA amplicon sequencing analysis was performed by Wuhan Maiwei Metabolic Biotechnology Co., Ltd., Wuhan, China. Ten intact intestinal tissue samples were randomly selected from each experimental group and subjected to thorough grinding and pulverization. Genomic DNA was extracted from the samples using the cetyltrimethylammonium bromide (CTAB) method, and the purity and concentration of DNA were examined by agarose gel electrophoresis. An appropriate amount of DNA sample was diluted to a final concentration of 1 ng/μL with sterile water for subsequent use. Using the diluted genomic DNA as the template, PCR amplification was carried out with the specific primers 515F and 806R targeting the V4 hypervariable region of the 16S rRNA gene, together with barcode-specific primers, Phusion^®^ High-Fidelity PCR Master Mix with GC Buffer (New England Biolabs, Ipswich, MA, USA), and high-efficiency, high-fidelity DNA polymerase. After verification by 2% agarose gel electrophoresis, the qualified PCR products were purified with magnetic beads, quantified by microplate reader-based quantification, pooled in an equimolar ratio, and rechecked by 2% agarose gel electrophoresis. The target bands were finally recovered using a Qiagen gel extraction kit. Sequencing libraries were constructed using the TruSeq^®^ DNA PCR-Free Sample Preparation Kit, and the qualified libraries after dual quantification by Qubit and quantitative real-time PCR (qPCR) were sequenced on the Illumina NovaSeq6000 platform. Raw reads were demultiplexed according to barcode and primer sequences, followed by trimming of barcode and primer regions. High-quality paired-end reads were obtained by filtering raw data with fastp software (v0.22.0; https://github.com/OpenGene/fastp, accessed on 30 October 2023) under the following criteria: automatic detection and removal of adapter sequences; removal of reads with ≥15 N-bases; removal of reads with> 50% low-quality bases (quality score ≤ 20); deletion of sequences with an average quality score < 20 within a 4-base sliding window; trimming of poly-G tails at the read ends; and exclusion of reads shorter than 150 bp. The high-quality paired-end reads were merged into clean tags using FLASH software (v1.2.11; http://ccb.jhu.edu/software/FLASH/, accessed on 30 October 2023). Chimeric sequences were detected and removed by aligning clean tags against the species annotation database with vsearch software (v2.22.1; https://github.com/torognes/vsearch/, accessed on 30 October 2023), thus obtaining the final effective tags. Taxonomic annotation of amplicon sequence variants (ASVs) was performed using Mothur software (v1.48) against the SILVA138.1 SSUrRNA database (http://www.arb-silva.de/, accessed on 30 October 2023) with a confidence threshold of 0.8–1. Taxonomic assignments were obtained, and the microbial community composition of each sample was analyzed at the phylum, class, order, family, genus, and species levels. Multiple sequence alignment of all ASV representative sequences was conducted using MAFFT software (v7.520; https://mafft.cbrc.jp/alignment/software/, accessed on 30 October 2023) to determine phylogenetic relationships. All samples were then rarefied to the minimum sequencing depth among samples for normalization. Based on the normalized data, alpha diversity indices including Observed ASV, Chao1, Shannon, Simpson, ACE, and PD_whole_tree were calculated using the phyloseq (v1.40.0) and vegan (v2.6.2) packages in R software (v4.2.0).

### 2.5. Determination of Metabolites in Serum

The determination of serum metabolites was entrusted to Wuhan Maiwei Metabolic Biotechnology Co., Ltd., Wuhan, China. Five fish serum samples were randomly selected from each experimental group; after thawing, the samples were vortexed for 10 s to mix evenly, and 50 μL of each sample was accurately pipetted into a correspondingly numbered centrifuge tube. Then, 300 μL of 20% acetonitrile–methanol extraction solution containing an internal standard was added to these centrifuge tubes, followed by vortexing for 3 min. Subsequently, the mixture was centrifuged at 12,000 rpm for 10 min at 4 °C. After centrifugation, 200 μL of the supernatant was aspirated into a new container and allowed to stand at −20 °C for 30 min. Afterwards, the mixture was centrifuged again at 12,000 rpm for 3 min at 4 °C, and 180 μL of the final supernatant was transferred into the inner tube of a sample vial for liquid chromatography-tandem mass spectrometry (LC-MS/MS) analysis. The serum samples from different groups were labeled as SCK0, SCA0, SCK12, and SCA12, respectively. For the chromatographic conditions, a Waters ACQUITY Premier HSS T3 column (1.8 µm, 2.1 mm × 100 mm) was used; mobile phase A was 0.1% formic acid aqueous solution, and mobile phase B was 0.1% formic acid acetonitrile solution; the column temperature was 40 °C, the flow rate was 0.4 mL/min, and the injection volume was 4 μL. For the mass spectrometric conditions, an AB SCIEX TripleTOF 6600+ mass spectrometer (Foster City, CA, USA) was employed with the electrospray ionization negative mode (ESI^−^), and the specific parameters were as follows: detection duration of 10 min; ion spray voltage of −4000 V, ion source temperature of 450 °C; ion source gas 1 pressure of 50 psi, ion source gas 2 pressure of 60 psi, curtain gas pressure of 35 psi; declustering potential of −60 V, MS1 collision energy of −10 V, MS2 collision energy of −30 V, collision energy spread of 15 V; MS1 mass-to-charge ratio (*m*/*z*) scan range of 50–1000 Da, MS2 *m*/*z* scan range of 25–1000 Da; MS1 accumulation time of 0.2 s, MS2 accumulation time of 0.04 s; number of candidate ions of 18; target ion exclusion rule: always exclude, with a duration of 3 s, triggered after 3 occurrences. For data analysis, ProteoWizard software (version 3.0.22335; https://proteowizard.sourceforge.io, accessed on 27 October 2023) was used to convert the raw mass spectrometric data into mzXML format, and the XCMS program was applied for peak extraction, peak alignment, and retention time correction. The support vector regression (SVR) method was used to correct the peak areas, and peaks with a missing rate > 50% across samples in each group were filtered out. The non-targeted metabolome identification was prioritized as follows: company-built local high-resolution database ˃ online databases [Metlin (http://metlin.scripps.edu/index.php, accessed on 27 October 2023), HMDB (https://hmdb.ca/, accessed on 27 October 2023), KEGG (https://www.kegg.jp/, accessed on 27 October 2023), Mona (https://mona.fiehnlab.ucdavis.edu/, accessed on 27 October 2023), MassBank (http://www.massbank.jp/, accessed on 27 October 2023), etc.] ˃ MetDNA-constructed derived database ˃ PubChem and ChemSpider databases. Only metabolites with an identification score ≥ 0.5 were retained for subsequent analysis.

### 2.6. Statistical Analysis

One-way ANOVA (One-way Analysis of Variance) was conducted and charts were created using SPSS 26.0, Microsoft Excel 2010, and R software (version 4.1.2). The data were presented in the form of mean ± standard error (mean ± SE). Statistical significance was indicated by a *p*-value less than 0.05.

## 3. Results

### 3.1. Microbiome Changes

#### 3.1.1. Species Diversity Curve and Venn Diagram

**Figure 1:**
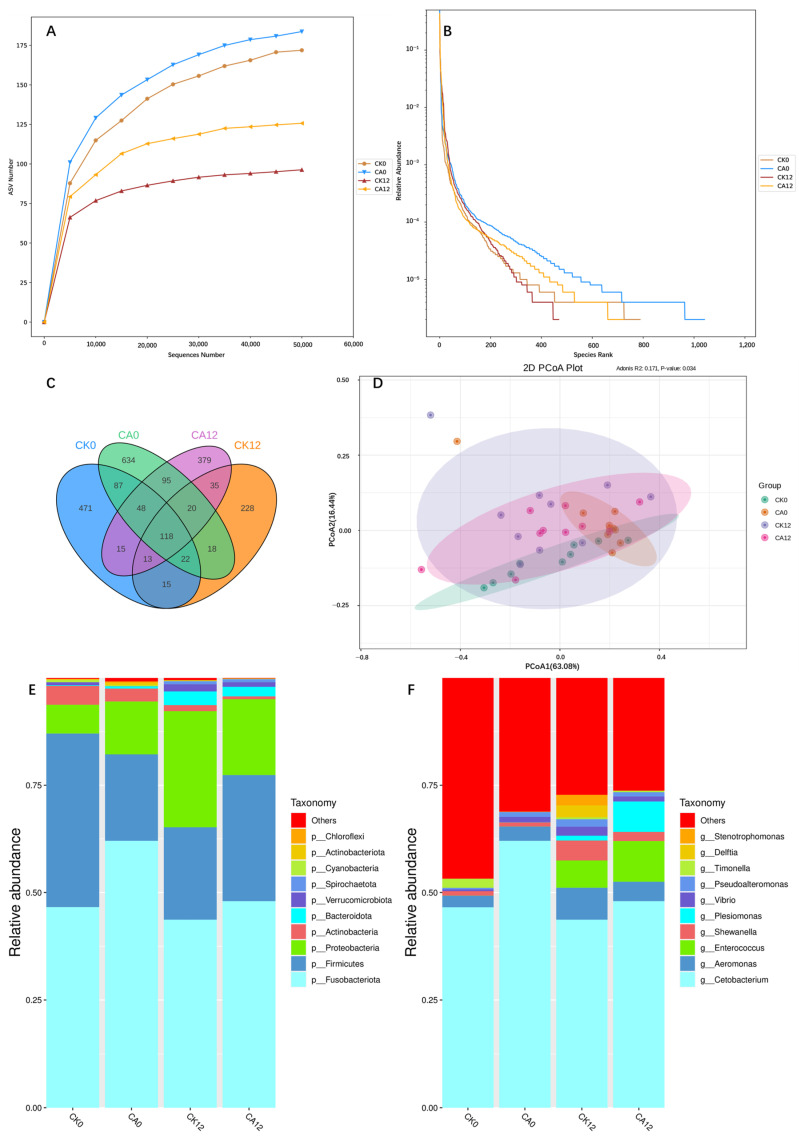
Analysis of Intestinal Microbial Composition: (**A**) Rarefaction curve based on ASV (Horizontal axis: Number of sequencing reads, Vertical axis: Number of amplicon sequence variants); (**B**) Rank abundance curve based on ASV (Horizontal axis: Ranked order of species by abundance, Vertical axis: Relative abundance of species, logarithmic scale); (**C**) Venn diagram based on ASV (Non-overlapping areas: Number of unique ASVs in the corresponding group, Overlapping areas: Number of shared ASVs between groups); (**D**) PCoA (Principal Co-ordinates Analysis) analysis based on ASV-derived weighted UniFrac distance (the horizontal axis represents one principal component, while the vertical axis represents another; the percentage indicates the contribution rate of the corresponding principal component to sample variation); (**E**) Phylum-level structural composition of intestinal microbiota across different groups (Horizontal axis: Experimental groups, Vertical axis: Relative abundance of microbial phyla); (**F**) Genus-level structural composition of intestinal microbiota across different groups (*n* = 10) (Horizontal axis: Experimental groups, Vertical axis: Relative abundance of microbial genera) ^1^. ^1^ CK0 and CA0: the samples before challenge test; CK12 and CA12: the samples after challenge for 12 h of CK and CA group, respectively. The Venn diagram was utilized to depict the shared and unique microbial species among different groups; the Venn diagram based on ASV analysis for each experimental group is shown in Figure 1C. The ASV counts for CK0, CA0, CK12, and CA12 samples were 789, 1042, 469, and 723, respectively. The CK0 and CA0 groups share 275 ASVs, making up 34.85% of CK0’s total and 26.39% of CA0’s. Between CK0 and CK12, 168 ASVs are shared, accounting for 21.29% of CK0’s and 35.82% of CK12’s totals. Similarly, CA0 and CA12 share 281 ASVs, representing 26.97% of CA0’s and 38.87% of CA12’s total ASVs.

The ASV (Amplicon Sequence Variant)-based rarefaction curve and rank abundance curve are shown in Figure 1. The rarefaction curve serves to evaluate the extent of sequencing. As shown in Figure 1A, the rarefaction curves of each group gradually plateaued with the progression of sequencing, indicating that the sequencing depth was sufficient to comprehensively represent the species diversity of the samples. Based on the sorting of ASVs from highest to lowest abundance, the rank abundance curve depicts the relationship between species abundance and their rank. As depicted in Figure 1B, the rank abundance curves of the various experimental groups showed that the ASV abundance in the CA group was higher compared to the CK group before the challenge test, and similar results were observed post-challenge. Notably, the curves for the CK and CA groups at different timepoints exhibited a smoother pattern, indicating a more even distribution of ASV relative abundances.

#### 3.1.2. Principal Co-Ordinates Analysis (PCoA)

The PCoA analysis diagram of each experimental group at different timepoints is shown in Figure 1D. The addition of carvacrol to the feed significantly altered the intestinal microbial structure. After challenge with *A. hydrophila*, the intestinal microbial community structure was disrupted, and the degree of disruption was higher between the CK groups compared to the CA groups.

#### 3.1.3. Statistical Analysis of Intestinal Microbial α-Diversity

The α-diversity statistical analysis of intestinal microbiota in Pengze crucian carp dietary with carvacrol supplementation is presented in Table 2. The results indicate that there was no significant difference (*p* > 0.05) in the observed ASV count between the CA group and CK group. The Chao1 (Chao1 estimator) and ACE (Abundance-based Coverage Estimator) indices, which are used to describe the richness of microbial communities, showed no significant changes (*p* > 0.05). After exposure to the pathogens, the microbial diversity indices, including Observed ASV, Chao1, and ACE, all declined in both the CA and CK groups. There was no significant difference observed between CA12 and CA0 (*p* > 0.05), whereas a significant difference was noted between CK12 and CK0 (*p* < 0.05).

#### 3.1.4. Analysis of Intestinal Microbial Composition

The composition of intestinal microbiota in each group at the phylum level is presented in Figure 1E and Table 3. Statistical analysis revealed significant differences in the relative abundances of seven bacterial phyla: *Fusobacteriota*, *Firmicutes*, *Proteobacteria*, *Actinobacteria*, *Bacteroidota*, *Verrucomicrobiota*, and *Cyanobacteria*. Notably, *Fusobacteriota* emerged as the dominant phylum. The addition of carvacrol significantly decreased the relative abundance of *Firmicutes* (*p* < 0.05). However, 12 h after challenge with *A. hydrophila*, the impact of carvacrol on the relative abundances of intestinal microbiota at the phylum level was not significant (*p* > 0.05).

The composition of intestinal microbiota at the genus level across various experimental groups are illustrated in Figure 1F and Table 4. Among the top 10 most abundant genera, significant differences (*p* < 0.05) were observed in the relative abundances of seven genera: *Cetobacterium*, *Aeromonas*, *Enterococcus*, *Shewanella*, *Plesiomonas*, *Vibrio*, and *Timonella*. Notably, *Cetobacterium* emerged as the dominant genus. Following the addition of carvacrol, the relative abundance of *Cetobacterium* increased by 33.25%, while that of *Timonella* significantly decreased (*p* < 0.05). Post-challenge, the relative abundance of *Plesiomonas* in the CA group significantly increased (*p* < 0.05). Intriguingly, the relative abundances of *Aeromonas*, *Shewanella* and *Vibrio* were significantly increased in the CK group after being exposed to *A. hydrophila* (*p* < 0.05), while no significant changes were observed in the CA group. In contrast, the levels of *Enterococcus* and *Plesiomonas* in the CA group significantly increased (*p* < 0.05), and the abundance of the potentially beneficial bacterium *Cetobacterium* increased by 33.25%.

#### 3.1.5. Functional Annotation of Intestinal Microbiota

**Figure 2:**
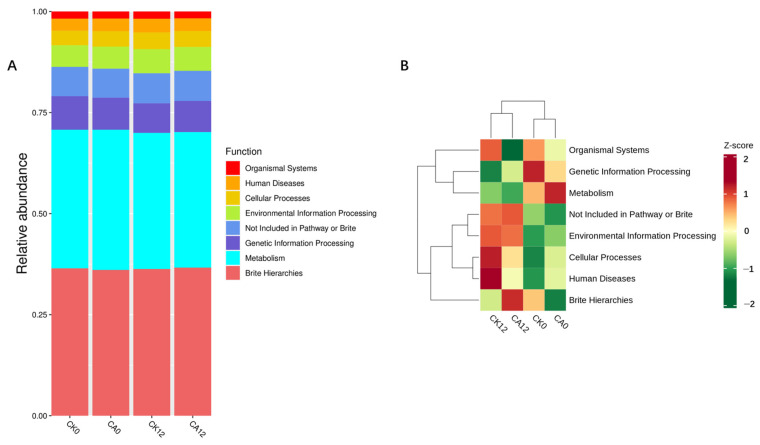
Functional annotation of intestinal microbiota based on ASV using PICRUSt2 ^1^. (**A**) Bar plot of relative abundance of functional annotations; (**B**) Clustered heatmap of relative abundance of functional annotations. ^1^ CK0 and CA0: the samples before challenge test; CK12 and CA12: the samples after challenge for 12 h of CK and CA group, respectively.

Based on the functional annotations and relative abundance information of samples, relative abundance bar charts and cluster heatmaps are shown in Figure 2 and Table 5. Carvacrol had no significant impact on the functional classification of gut microbiota across experimental groups (*p* > 0.05). However, 12 h post-challenge, the organismal systems in the CA group were significantly lower than those in the CK group (*p* < 0.05).

### 3.2. Changes in Blood Metabolomics

#### 3.2.1. General Overview of Metabolites in the Blood of Pengze Crucian Carp

**Figure 3:**
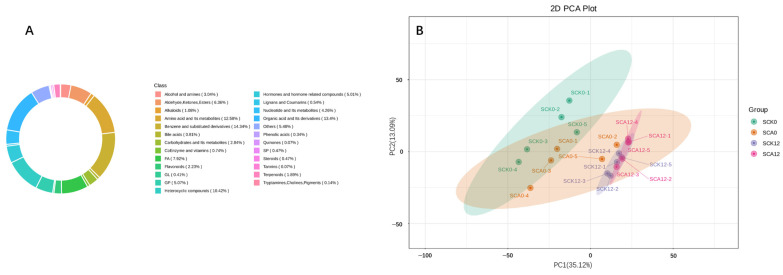
Analysis of serum metabolite composition: (**A**) Circular plot of serum metabolite category composition from mass spectrometry (MS) analysis (Values in parentheses: Proportion of each class relative to total detected metabolites); (**B**) PCA (Principal Component Analysis) of various experimental groups in negative ion mode (*n* = 5) ^1^. ^1^ SCK0 and SCA0: the serum samples before challenge test; SCK12 and SCA12: the serum samples after challenge for 12 h of the CK and CA group, respectively.

A total of 2792 metabolites were detected from 20 samples in this experiment, with 2507 of them confirmed at the secondary identification level. Among these, 1423 metabolites were detected in the positive ion mode of mass spectrometry (MS), while 1369 were detected in the negative ion mode. The circular diagram depicting the composition of metabolite categories is presented in Figure 3A.

#### 3.2.2. Overall Differences in Blood Metabolites of Different Treatments

To preliminarily understand the separation trend of metabolites among the experimental groups, we conducted a Principal Component Analysis (PCA), as shown in Figure 3B. The addition of carvacrol resulted in a separation trend in serum metabolites. Challenge with pathogens significantly altered the composition of serum metabolites, with a notable separation observed post-challenge in the CK group compared to the CA group.

#### 3.2.3. Metabolic Differences Among Different Treatments

**Figure 4:**
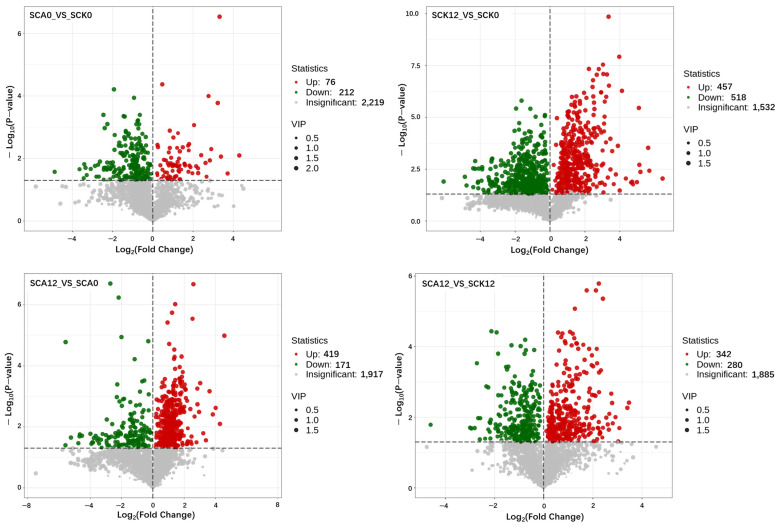
Volcano plot of differential metabolites between different experimental groups (*n* = 5) (Horizontal axis (Log_2_(Fold Change)): Log_2_-transformed fold change of metabolite abundance between the two groups, Vertical axis (−log_10_(*p*-value)): Negative logarithm of the *p*-value) ^1^. ^1^ SCK0 and SCA0: the serum samples before challenge test; SCK12 and SCA12: the serum samples after challenge for 12 h of the CK and CA group, respectively.

Volcano plots are primarily utilized to visually present the differences in the relative abundances of metabolites across different samples as well as their statistical significance. The comparison of metabolic differences among various experimental groups is illustrated in Figure 4. Compared to the CK0 samples, 76 serum metabolites were significantly upregulated (*p* < 0.05), while 212 were significantly downregulated (*p* < 0.05) in the CA0 samples of Pengze crucian carp. A total of 457 significantly increased metabolites (*p* < 0.05), and 518 significantly decreased metabolites (*p* < 0.05) were observed after being challenged with *A. hydrophila* in the CK group (CK12 vs. CK0). In contrast, in the carvacrol-treated group post-challenge, 419 significantly increased metabolites (*p* < 0.05) and 171 significantly decreased metabolites (*p* < 0.05) were detected after being challenged with *A. hydrophila* in the CA group (CA12 vs. CA0). When comparing the metabolites between CA and CK group at 12 h post-challenge, 342 serum metabolites were significantly upregulated (*p* < 0.05), while 280 were significantly downregulated (*p* < 0.05).

**Figure 5:**
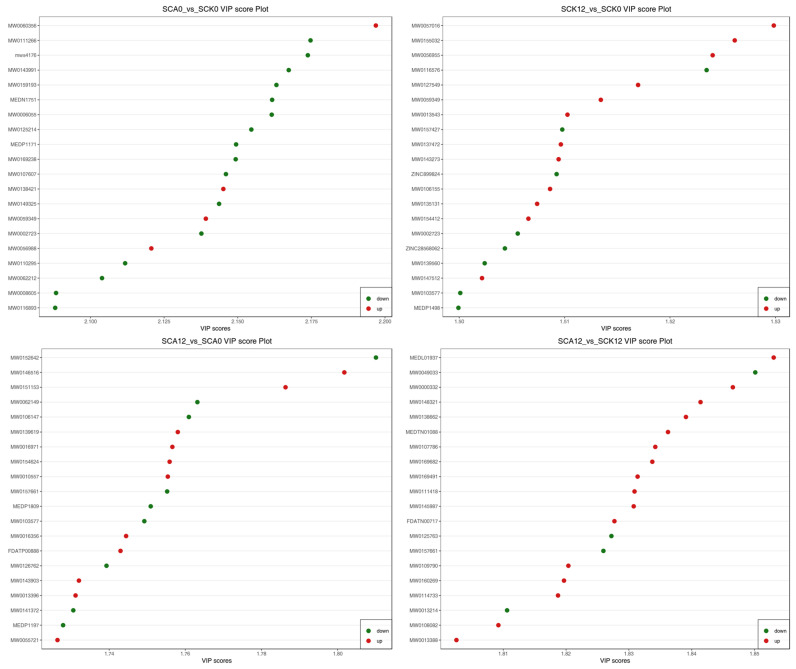
VIP values plot of differential metabolites ^1^. Note: The horizontal axis represents VIP values, while the vertical axis represents differential metabolites. Red indicates upregulated differential metabolites, and green indicates downregulated differential metabolites (Horizontal axis: VIP (Variable Importance in Projection) value (reflects the contribution of each metabolite to group separation; higher values indicate greater importance), Vertical axis: Unique IDs of differential metabolites) ^1^. ^1^ SCK0 and SCA0: the serum samples before challenge test; SCK12 and SCA12: the serum samples after challenge for 12 h of the CK and CA group, respectively.

A VIP plot was generated by selecting the top 20 metabolites with the highest VIP scores from each group in the OPLS-DA model. As shown in Figure 5, carvacrol significantly downregulated 16 metabolic substances, including Gramine, Alanylphenylalanine, Tyrosyl-tryptophan, and 2-Hexyldecanoic acid (*p* < 0.05), while notably upregulating four metabolic substances belonging to Phosphatidylethanolamine (PE), Phosphatidylcholine (PC), and Heterocyclic compounds (*p* < 0.05) (CA0 vs. CK0). Following exposure to pathogens, 12 significantly upregulated metabolic substances in the CK group (*p* < 0.05), and 11 upregulated substances in the CA group (*p* < 0.05) were observed. Compared to the CK group, 16 serum metabolic substances were upregulated after being challenged with *A. hydrophila* (*p* < 0.05).

**Figure 6:**
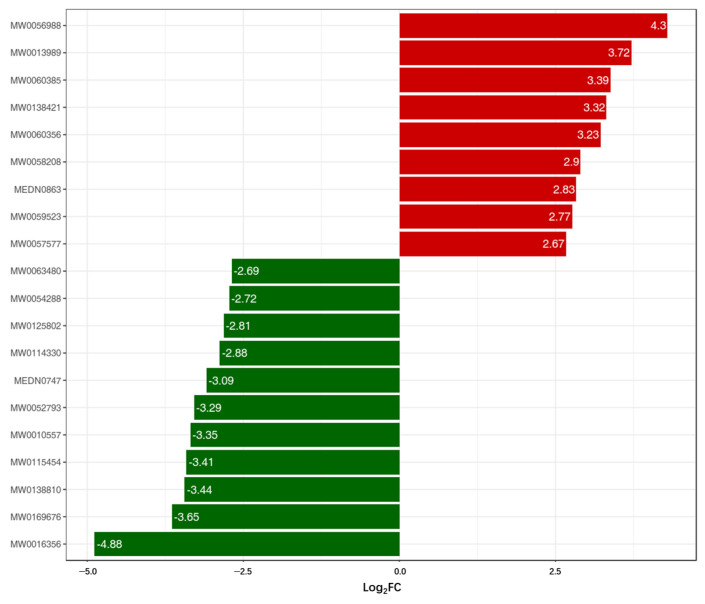
Log2FC of differential metabolites between the SCA0 group and SCK0 group (Horizontal axis: Log_2_FC (Log_2_-transformed fold change in metabolite abundance between SCA0 and SCK0; positive values = higher abundance in SCA0; negative values = higher abundance in SCK0), Vertical axis: Unique IDs of differential metabolites) ^1^. ^1^ SCK0 and SCA0: the serum samples before challenge test. Red bars represent positive Log_2_FC values, and green bars represent negative Log_2_FC values.

In these comparisons, we focused on the top 20 different metabolites between the CK and CA group before the challenge test. Based on the quantitative metabolite information from the SCA0 and SCK0 groups, a bar chart was generated to illustrate the top 20 metabolites exhibiting the most significant fold changes (Figure 6). Among these different metabolites, nine glycerophospholipids and bile acids, including 3β-Hydroxy-5-cholenoic acid and 25-Hydroxycholesterol, exhibited increased levels. Conversely, eleven substances, such as Dodecanoic acid and Valeraldehyde dibutyl acetal, demonstrated a decreased tendency.

#### 3.2.4. KEGG Enrichment Analysis of Differential Metabolites Across Treatments

**Figure 7:**
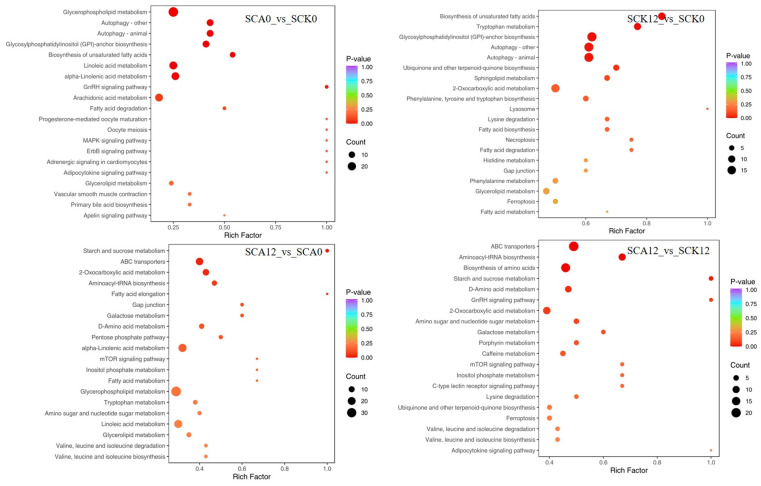
KEGG enrichment analysis of differential metabolites (Horizontal axis (Rich Factor): Ratio of differential metabolites mapped to a pathway, relative to all metabolites annotated to that pathway. A higher value indicates stronger pathway enrichment. Vertical axis: Names of significantly enriched KEGG metabolic pathways) ^1^. ^1^ SCK0 and SCA0: the serum samples before challenge test; SCK12 and SCA12: the serum samples after challenge for 12 h of the CK and CA group, respectively.

A KEGG (Kyoto Encyclopedia of Genes and Genomes) pathway enrichment analysis was performed on the top 20 pathways ranked by *p*-value based on differential metabolite data, as presented in Figure 7. Compared to SCK0, SCA0 exhibited the most significant enrichment of serum differential metabolites in pathways such as glycerophospholipid metabolism, linoleic acid metabolism, arachidonic acid metabolism, and α-linolenic acid metabolism, among others. Following the challenge, SCA12 displayed an increased number of differential metabolites compared to SCK12 in pathways including ABC transporters, biosynthesis of amino acids, biosynthesis of cofactors, 2-oxocarboxylic acid metabolism, aminoacyl-tRNA biosynthesis, nucleotide metabolism, D-amino acid metabolism, purine metabolism, pyrimidine metabolism, and mTOR signaling pathway.

## 4. Discussion

### 4.1. Impact of Carvacrol on the Intestinal Microbial Composition of Pengze Crucian Carp

Stable intestinal microbiota play a pivotal role in the growth and development of fish [21], facilitating digestion and absorption in the host’s gastrointestinal tract on the one hand [22], and maintaining overall health by establishing a biofilm barrier through the colonization of beneficial microbial flora in the intestine on the other hand [23]. In this study, *Fusobacteriota* and *Firmicutes* were identified as the dominant phyla in the intestinal microbiota of Pengze crucian carp. At the phylum level, carvacrol significantly reduced the abundance of *Firmicutes* while increasing the level of *Fusobacteriota* by 28.87%, albeit statistically insignificant. *Firmicutes* in fish intestines are crucial for food fermentation and nutrient absorption; however, our previous research indicated no notable impact of carvacrol on growth indicators of Pengze crucian carp [19]. Moreover, the functional annotation of intestinal microbiota in this experiment revealed no significant influence of carvacrol on intestinal functions such as metabolism and cellular processes. Hence, the increase in *Fusobacteriota* abundance may be partially attributed to the decrease in *Firmicutes* proportion. The growth in *Fusobacteriota* abundance is of particular interest to us. Although statistically non-significant, carvacrol supplementation in the feed of Pengze crucian carp enhanced the abundance of *Cetobacterium*, a genus belonging to the *Fusobacteriota* phylum, by 33.25%. *Cetobacterium* is ubiquitous in fish intestines [24,25]. Based on whole-genome sequencing, *Cetobacterium* possesses all genes required for folate synthesis (including folP, folC, folA, etc.) as well as those required for the conversion of microbial B12 into its coenzyme form [26]. A study by Qi et al. on zebrafish revealed that vitamin B12 produced by intestinal *Cetobacterium* is vital for zebrafish to resist *A. hydrophila* infection and significantly improves the stability of the intestinal microbial ecological network [27].

On the other hand, the statistical analysis of α-diversity of intestinal microbiota in Pengze crucian carp showed no significant effect from the addition of carvacrol in this experiment. However, comparisons before and after pathogen challenge revealed that the stability of intestinal microbiota diversity was superior in the CA group compared to CK. Furthermore, after the pathogen challenge, the relative abundance of *A. hydrophila* in the intestine decreased by 39.19% in the CA group, indicating that carvacrol enhanced the environmental resistance of the intestinal microbiota in Pengze crucian carp, improved its intestinal microbial barrier function, and decreased the abundance of *A. hydrophila*. There are few studies solely focused on the impact of carvacrol on intestinal microbiota diversity. However, carvacrol is a major component of oregano essential oil. Dang et al. found that feeding rainbow trout with oregano essential oil increased the levels of beneficial intestinal bacteria such as *Streptococcus lutetiensis*, *Oscillospirales*, and *Methylobacterium-Methylorubrum*, while reducing the levels of potentially harmful intestinal bacteria such as *Mycoplasma*, *Morganellaceae*, and *Paenalcaligenes*. This treatment also enhanced the disease resistance of rainbow trout against *Aeromonas salmonicida* [28], aligning with the results of our experiment. The reasons for carvacrol enhancing the intestinal microbial barrier function may be multifaceted. On one hand, carvacrol disrupts bacterial membrane permeability by altering unsaturated fatty acid composition and inhibiting ATP synthesis [29], thereby inhibiting and killing exogenous pathogens such as *A. hydrophila* and *Edwardsiella tarda* [12,30]. This provides ecological niches for intestinal commensal bacteria and promotes the enhancement of intestinal microbiota stability [31], and consequently strengthens the intestinal biological barrier function. On the other hand, our previous research showed that carvacrol promotes the structural integrity of fish intestines [19], effectively improving the physical and structural barriers within the intestines. The role of carvacrol in enhancing the structural integrity of fish intestines may also be related to the increase in the content of *Cetobacterium*. It has been reported that the cell-free culture supernatant (CFCS) of *Cetobacterium* isolated from the intestine of crucian carp (*Carassius auratus*) exhibits functions such as scavenging DPPH, hydroxyl radicals, and superoxide anions, and enhances the resistance of zebrafish to *A. hydrophila* infection [32]. Meanwhile, studies have shown that carvacrol can, to a certain extent, enhance immune indicators such as myeloperoxidase activity and phagocytic activity in fish [33], thereby strengthening immune barrier function and boosting the host’s resistance to potential pathogenic bacteria.

### 4.2. Effect of Carvacrol on the Composition of Serum Metabolites in Crucian Carp

To further investigate the effect of carvacrol on the physiological metabolic processes of Pengze crucian carp, we conducted metabolomic analysis on the serum components of various experimental groups. The results of the Principal Component Analysis (PCA) indicated that carvacrol could affect the serum metabolites, with more pronounced changes observed in serum metabolites after pathogen challenge. The Kyoto Encyclopedia of Genes and Genomes (KEGG) pathway enrichment results revealed that the differential metabolites in the serum of Pengze crucian carp treated with carvacrol were mainly involved in multiple pathways such as glycerophospholipid metabolism, linoleic acid metabolism, arachidonic acid metabolism, alpha-linolenic acid metabolism, and glycosylphosphatidylinositol (GPI)-anchor biosynthesis. Among them, the most enriched pathway was glycerophospholipid metabolism. The Variable Importance in the Projection (VIP) values from the OPLS-DA model also demonstrated significant upregulation of Phosphatidylcholine (PC) and Phosphatidylethanolamine (PE) content in the serum of the carvacrol-treated group. PC is one of the major components of cell membranes. It serves as a hydrophobic barrier against the microbiota [34] and enhances intestinal mucus barrier function through a surface hydration-dependent adsorption process to mucins [35]. Further studies have demonstrated that exogenous supplementation of PC can increase the expression of caveolin-1 in cell membranes, thereby preventing changes in membrane permeability and cytoplasmic leakage induced by caveolin-1 downregulation, meanwhile, it can inhibit lipid peroxidation to protect cell membranes from damage [36]. On the other hand, PE is one of the key regulators of membrane fluidity in eukaryotic cells [37]. It assists in membrane protein folding and maintains membrane protein function; increased PE content activates phosphatidylethanolamine-binding protein (PEBP), which negatively regulates the cellular apoptotic process [38]. Combined with our previous observations, dietary supplementation of 600 mg/kg carvacrol improved intestinal morphological integrity and reduced the levels of the intestinal pro-inflammatory cytokines tumor necrosis factor-α (TNF-α) and interleukin-1β (IL-1β) [19]. Therefore, the upregulation of PC and PE observed in this study may be one of the metabolic bases underlying these morphological improvements.

Carvacrol exhibits close interconnections and influences among various fatty acid metabolic pathways such as linoleic acid metabolism, arachidonic acid metabolism, and alpha-linolenic acid metabolism, which are integral to essential fatty acid metabolism [39,40]. Furthermore, carvacrol can indirectly impact the host’s immune function through these fatty acid metabolic processes. In animals, linoleic acid (18:2n − 6) can be converted into arachidonic acid (20:4n − 6) [41], which is further catalyzed into various biologically active substances such as prostaglandins and leukotrienes through enzymes like cyclooxygenases (COX) and lipoxygenases (LOX), participating in inflammatory responses, immune regulation, and cellular signal transduction [42]. Additionally, carvacrol has been reported to exhibit potent anti-inflammatory effects by inhibiting the activity of COX and LOX enzymes [43,44], reducing the production of arachidonic acid metabolites and decreasing inflammatory responses [45]. Furthermore, both linoleic acid and α-linolenic acid are important components of cell membrane phospholipids, yet they compete at the enzymatic level, for instance, both rely on Δ6-desaturase for the initial desaturation reaction. High intake of linoleic acid may preferentially occupy key enzymes such as Δ6-desaturase, thereby inhibiting the metabolism and conversion of α-linolenic acid, leading to the decreased synthesis of ω-3 fatty acids like EPA and DHA [46], which regulate immune responses in the body. Multiple factors influence the regulation of immune function by carvacrol through fatty acid metabolism. Studies have reported that carvacrol possesses strong antioxidant capabilities, potentially mitigating oxidative stress during linoleic acid metabolism by reducing the generation or scavenging of free radicals [47]. Additionally, carvacrol can affect the activity of key enzymes involved in lipid metabolism, such as acyl-CoA synthetase, acyl-CoA dehydrogenase, and phosphatidylglycerophosphate synthase [48], thereby regulating the rate and direction of linoleic acid metabolism.

The results of this experiment demonstrate an impact of carvacrol on the levels of acidic compounds in serum, encompassing bile acids and organic acids. Specifically, the content of 3β-hydroxy-5-cholenoic acid, a primary bile acid, was increased by 13.14 times compared to the control group. Additionally, Asiatic acid levels rose by 4.74 times, prephenic acid by 2.23 times, 1,3,4-trihydroxy-5-oxocyclohexane-1-carboxylic acid by 2.41 times, and 8,11,14-eicosatrien-5-ynoic acid by 3.04 times, among others. Bile acids, as one of the end products of cholesterol metabolism, constitute a crucial component of bile. They not only facilitate the absorption of lipids in the intestinal tract of aquatic animals [49] but also exert notable effects on intestinal microbiota and immune function. Previous studies have shown that exogenous bile acid supplementation can enhance intestinal microbial diversity [50] and reduce the abundance of harmful bacteria [51], primarily by promoting the secretion of antimicrobial peptides by intestinal epithelial cells [52] and maintaining intestinal barrier function [53]. The bile acid receptors FXR and TGR5 regulate the expression of junction proteins, thereby decreasing the permeability of the animal intestinal wall and enhancing barrier capacity [54,55]. In this study, carvacrol promoted an elevation in serum levels of 3β-hydroxy-5-cholenoic acid, which can further be converted into chenodeoxycholic acid and lithocholic acid in vivo [56]. Organic acids in serum also modulate the immune function of animal organisms. For instance, Asiatic acid promotes autophagy to alleviate acute kidney injury (AKI) [57], induces apoptosis in breast cancer cells [58], and attenuates IL-6 expression and the phosphorylation of STAT3 and NF-κB, thereby inhibiting the production of inflammatory cells and cytokines [59], among other effects.

The addition of carvacrol to feed may also directly impact the immune function of Pengze crucian carp. The results of this experiment showed that before pathogenic challenge, all 12 of the serum metabolites related to the autophagy pathway (autophagy-animal) in the carvacrol treatment group were glycerophospholipids and glycerides, with 11 of them being upregulated. This indicates that carvacrol may not only indirectly enhance the immune capacity of Pengze crucian carp through fatty acid metabolism but also enhance the expression of autophagy signaling pathways in the organism. Autophagy, as a process of cellular degradation, identifies and eliminates pathogen-infected organelles or entire cells, thereby limiting the spread of pathogens. It is a hot topic in the field of health maintenance [60]. The enhancement of autophagy function by carvacrol without the need for fasting can be considered an important manifestation of its health-promoting properties [61]. After infection with pathogenic bacteria, serum metabolic differences in the control group were enriched in the autophagy signaling pathway, while those in the carvacrol-added group were enriched in the mTOR signaling pathway. Furthermore, compared to the control group, L-arginine and L-leucine in the serum metabolites of the carvacrol-added group were upregulated and enriched in the mTOR signaling pathway. In the non-fasting state, the mTOR signaling pathway inhibits autophagy by phosphorylating specific sites (Ser637 and Ser757) on the autophagy-regulating complex (ULK1 complex) and Ser258 on Atg13, thereby reducing the activity of autophagy-promoting kinases [62]. This suggests that the addition of carvacrol alone may promote autophagy in Pengze crucian carp. However, under the stress of *A. hydrophila*, carvacrol inhibits the autophagy process, potentially through the mTOR signaling pathway.

## 5. Conclusions

In conclusion, this study demonstrates that dietary carvacrol exerts dual protective effects in Pengze crucian carp through: (1) modulating intestinal microbial composition by suppressing Firmicutes proliferation while promoting beneficial *Cetobacterium* growth (33.25% increase), and enhancing microbial community stability and ecological resilience, which significantly reduces the proportion of *A. hydrophila*; and (2) systematically regulating serum metabolic homeostasis, particularly through acidic metabolite modulation and critical pathway alterations in lipid metabolism (glycerophospholipid, linoleic/α-linolenic acid pathways) and immune regulation (GPI-anchor biosynthesis, autophagy). Our findings provide evidence characterizing carvacrol’s regulatory effects on gut microbiota and systemic metabolism in aquatic species, offering valuable theoretical foundations for developing plant-derived immunostimulants in ecologically sustainable aquaculture systems.

## Figures and Tables

**Table 1:** Diet Formulation and Chemical Composition.

Ingredient Percentage (%)	CK0	CA0
Fish meal	14.00	14.00
Soyabean meal	26.00	26.00
Rapeseed meal	23.00	23.00
Corn grain	22.00	22.00
Soyabean oil	2.00	2.00
Fish oil	1.00	1.00
Coated lysine	0.50	0.50
Coated methionine	0.25	0.25
Vitamin mixture ^a^	0.50	0.50
Mineral mixture ^b^	0.50	0.50
Choline	0.50	0.50
CaH_2_PO_4_	1.50	1.50
Vitamin C phosphate ester	0.10	0.10
Carboxymethyl cellulose sodium	2.00	2.00
Microcrystalline cellulose	6.15	5.55
Microencapsulated carvacrol	0	0.60
Chemical composition (%)		
Crude protein	33.14	33.15
Crude lipid	4.82	4.81
Crude ash	5.75	5.76
Moisture	10.2	10.1

CK, basal diet group; CA, basal diet supplemented with 600 ppm MC group. Note: ^a^ Vitamin mix (per kg mix): vitamin A, 350,000 IU; vitamin D, 3,450,000 IU; vitamin E, 20 g; menadione, 7.5 g; thiamin, 10 g; riboflavin, 10 g; pyrido, xamine, 12 g; cobalamin, 20 mg; nicotinamide, 40 mg; folic acid, 3 g; calcium pantothenate, 30 g; biotin, 100 mg; ascorbic acid, 60 g; inositol, 60 g. ^b^ Mineral mix (per 100 g mix): NaH_2_PO_4_, 10 g; KH_2_PO_4_, 21.5 g; Ca(H_2_PO_4_)_2_·2H_2_O, 26.5 g; CaCO_3_, 10.5 g; Ca-lactate, 16.5 g; MgSO_4_·7H_2_O, 10 g; AlCl_3_·2H_2_O, 1.2 g; ZnSO_4_·7H_2_O, 0.511 g; Fe-citrate, 0.061 g; MnSO_4_· 4H_2_O, 0.143 g; KI, 0.058 g; CuCl_2_, 0.051 g; CoCl_2_·6H_2_O, 0.176 g; KCl, 2.8 g.

**Table 2:** Statistical Analysis of Intestinal Microbial α-diversity (*n* = 10) ^1^.

Treatment	Observed ASV	Chao1	ACE	Shannon	Simpson	PD (Phylogenetic Diversity) Whole Tree
CK0	173.22 ± 21.09 ^a^	175.78 ± 21.20 ^a^	180.95 ± 21.68 ^a^	1.80 ± 0.11	0.69 ± 0.03	16.14 ± 1.40 ^ab^
CA0	184.50 ± 32.83 ^a^	187.65 ± 33.47 ^a^	192.58 ± 34.39 ^a^	1.89 ± 0.29	0.64 ± 0.04	17.87 ± 2.43 ^a^
CK12	95.90 ± 5.33 ^b^	96.54 ± 5.33 ^b^	97.77 ± 5.33 ^b^	2.11 ± 0.13	0.75 ± 0.04	11.41 ± 0.66 ^c^
CA12	126.60 ± 15.38 ^ab^	128.58 ± 15.54 ^ab^	131.37 ± 15.89 ^ab^	2.02 ± 0.16	0.72 ± 0.04	13.07 ± 1.23 ^bc^

^1^ CK0 and CA0: the samples before challenge test; CK12 and CA12: the samples after challenge for 12 h of CK and CA group, respectively. Different lowercase letters (a, b, c) within the same column indicate significant differences between groups (*p* < 0.05), while the same letter indicates no significant difference (*p* > 0.05).

**Table 3:** The Relative Abundance of Intestinal Microorganism on The Phylum Level (*n* = 10) ^1^.

Phylum	CK0	CA0	CK12	CA12
*Fusobacteriota*	46.59 ± 5.85 ^ab^	62.04 ± 6.43 ^a^	43.66 ± 6.76 ^b^	47.97 ± 5.64 ^ab^
*Firmicutes*	40.45 ± 4.94 ^a^	20.14 ± 1.43 ^b^	21.55 ± 4.40 ^b^	29.39 ± 5.37 ^ab^
*Proteobacteria*	6.70 ± 1.10 ^b^	12.32 ± 4.46 ^b^	27.06 ± 7.03 ^a^	17.68 ± 2.95 ^ab^
*Actinobacteria*	4.40 ± 1.45 ^a^	3.01 ± 1.87 ^ab^	1.39 ± 0.36 ^ab^	0.66 ± 0.29 ^b^
*Bacteroidota*	0.15 ± 0.09 ^c^	0.51 ± 0.39 ^bc^	3.21 ± 1.06 ^a^	2.23 ± 0.81 ^ab^
*Verrucomicrobiota*	0.71 ± 0.50 ^ab^	0.04 ± 0.02 ^b^	1.68 ± 0.64 ^a^	1.06 ± 0.62 ^ab^
*Spirochaetota*	0.00 ± 0.00	0.00 ± 0.00	0.70 ± 0.55	0.72 ± 0.59
*Actinobacteriota*	0.199 ± 0.06	0.62 ± 0.44	0.06 ± 0.04	0.05 ± 0.03
*Cyanobacteria*	0.50 ± 0.188 ^a^	0.21 ± 0.11 ^a^	0.14 ± 0.049 ^ab^	0.09 ± 0.03 ^b^
*Chloroflexi*	0.07 ± 0.03	0.27 ± 0.17	0.05 ± 0.03	0.02 ± 0.01
Others	0.22 ± 0.06	0.82 ± 0.60	0.51 ± 0.34	0.12 ± 0.03

^1^ CK0 and CA0: the samples before challenge test; CK12 and CA12: the samples after challenge for 12 h of CK and CA group, respectively. Different lowercase letters (a, b, c) within the same column indicate significant differences between groups (*p* < 0.05), while the same letter indicates no significant difference (*p* > 0.05).

**Table 4:** The Relative Abundance of Intestinal Microorganism on The Genus Level (*n* = 10) ^1^.

Genus	CK0	CA0	CK12	CA12
*Cetobacterium*	46.55 ± 5.86 ^ab^	62.03 ± 6.43 ^a^	43.65 ± 6.76 ^b^	47.96 ± 5.64 ^ab^
*Aeromonas*	2.69 ± 0.80 ^b^	3.33 ± 0.79 ^b^	7.45 ± 1.91 ^a^	4.53 ± 1.19 ^ab^
*Enterococcus*	0.03 ± 0.02 ^b^	0.01 ± 0.01 ^b^	6.36 ± 2.13 ^ab^	9.49 ± 5.66 ^a^
*Shewanella*	1.03 ± 0.47 ^b^	0.97 ± 0.25 ^b^	4.65 ± 1.62 ^a^	2.13 ± 0.62 ^ab^
*Plesiomonas*	0.00 ± 0.00 ^b^	0.04 ± 0.03 ^b^	1.11 ± 0.79 ^b^	7.09 ± 2.41 ^a^
*Vibrio*	0.41 ± 0.28 ^b^	1.24 ± 0.54 ^ab^	2.10 ± 0.79 ^a^	1.17 ± 0.39 ^ab^
*Pseudoalteromonas*	0.36 ± 0.25	1.13 ± 0.478	1.74 ± 0.71	1.00 ± 0.33
*Delftia*	0.00 ± 0.00	0.00 ± 0.00	2.79 ± 2.41	0.00 ± 0.00
*Timonella*	2.12 ± 0.98 ^a^	0.04 ± 0.03 ^b^	0.44 ± 0.26 ^b^	0.35 ± 0.21 ^b^
*Stenotrophomonas*	0.01 ± 0.01	0.04 ± 0.02	2.47 ± 2.03	0.00 ± 0.00
Others	46.79 ± 5.88 ^a^	31.18 ± 6.01 ^b^	27.24 ± 3.47 ^b^	26.27 ± 4.36 ^b^

^1^ CK0 and CA0: the samples before challenge test; CK12 and CA12: the samples after challenge for 12 h of CK and CA group, respectively. Different lowercase letters (a, b) within the same column indicate significant differences between groups (*p* < 0.05), while the same letter indicates no significant difference (*p* > 0.05).

**Table 5:** Relative Abundance of Functional Annotations from ASV-Based PICRUSt2 (*n* = 10) ^1^.

Genus	CK0	CA0	CK12	CA12
Brite Hierarchies	36.45 ± 0.10	36.05 ± 0.41	36.28 ± 0.16	36.63 ± 0.07
Metabolism	34.30 ± 0.13 ^ab^	34.68 ± 0.28 ^a^	33.68 ± 0.21 ^bc^	33.53 ± 0.24 ^c^
Genetic Information Processing	8.30 ± 0.06 ^a^	7.92 ± 0.22 ^ab^	7.31 ± 0.29 ^b^	7.69 ± 0.16 ^ab^
Not Included in Pathway or Brite	7.25 ± 0.04 ^b^	7.19 ± 0.03 ^b^	7.45 ± 0.09 ^a^	7.46 ± 0.08 ^a^
Environmental Information Processing	5.34 ± 0.03 ^b^	5.46 ± 0.05 ^b^	5.95 ± 0.17 ^a^	5.92 ± 0.23 ^a^
Cellular Processes	3.60 ± 0.05 ^b^	3.82 ± 0.11 ^ab^	4.16 ± 0.18 ^a^	3.93 ± 0.09 ^ab^
Human Diseases	2.98 ± 0.03	3.13 ± 0.18	3.37 ± 0.17	3.15 ± 0.06
Organismal Systems	1.79 ± 0.02 ^ab^	1.75 ± 0.03 ^ab^	1.80 ± 0.04 ^a^	1.70 ± 0.03 ^b^

^1^ CK0 and CA0: the samples before challenge test; CK12 and CA12: the samples after challenge for 12 h of CK and CA group, respectively. Different lowercase letters (a, b, c) within the same column indicate significant differences between groups (*p* < 0.05), while the same letter indicates no significant difference (*p* > 0.05).

## Data Availability

The original contributions presented in this study are included in the article Further inquiries can be directed to the corresponding author.
